# Autotransplantation of Mandibular Third Molar: A Case Report

**DOI:** 10.1155/2012/629180

**Published:** 2012-12-17

**Authors:** Pabbati Ravi kumar, Mandava Jyothi, Kantheti Sirisha, Khushboo Racca, Chalasani Uma

**Affiliations:** Department of Conservative Dentistry and Endodontics, GITAM Dental College and Hospital, Visakhapatnam 530045, Andhra Pradesh, India

## Abstract

Autogenous transplantation is a feasible, fast, and economical option for the treatment of nonsalvageable teeth when a suitable donor tooth is available. This paper presents successful autotransplantation of a mature mandibular left third molar (38) without anatomical variances is used to replace a mandibular left second molar (37). The mandibular second molar was nonrestorable due to extensive root caries and resorption of distal root. After extraction of mandibular second and third molars, root canal therapy was done for the third molar extraorally, and the tooth was reimplanted into the extracted socket of second molar site. After one year, clinical and radiographic examination revealed satisfactory outcome with no signs or symptoms suggestive of pathology. In selected cases, autogenous tooth transplantation, even after complete root formation of the donor tooth, may be considered as a practical treatment alternative to conventional prosthetic rehabilitation or implant treatment.

## 1. Introduction

The earliest report of tooth transplantation involves slaves in ancient Egypt, who were forced to give their teeth to their pharaohs [1]. This type of dental surgical intervention was documented first by Abulcassis, in 1050; however, only in 1564, the French dentist Ambroise Paré performed the first recorded surgery with details about tooth bud transplantation. In 1956, a transplantation technique for molars was described, and until today, the general guidelines of this surgical technique are practically the same. Not withstanding, some techniques have been developed aiming to improve the prognosis, such as two-stage transplantation and prototyping [2]. 

Tooth transplantation can be classified into autogenous (where a tooth/tooth bud from one socket is inserted into another socket in the same person), homogenous (if the donation is performed by a person of the same specie of the receptor), and heterogeneous (if the donor is from a different specie of the receptor) [2].   Autogenous tooth transplantation or autotransplantation is the surgical transplantation of a vital or endodontically treated tooth from its original location in the mouth to another site in the same individual [1]. 

Tooth transplantation is a possible treatment option for the replacement of extracted permanent tooth that is malformed or involved with carious destruction or traumatic injury. Despite the widespread use of dental implants, autogenous tooth transplantation is frequently performed to replace missing mature teeth [3]. The main advantages of this procedure are alterations in the development of the maxilla and mandibular alveolar bone can be avoided, and it constitutes a viable method due to high success rate and relatively low cost compared to the traditional methods of rehabilitation such as osseointegrated implants [2]. Successful transplantation depends on specific requirements of the patient, the donor tooth, and the recipient site.

### 1.1. Case Selection Criteria

Candidates must be in good health, able to follow postoperative instructions and should be available for follow-up visits. They should also demonstrate an acceptable level of oral hygiene and be amenable to regular dental care. Most importantly, the patients must have a suitable recipient site and donor tooth. Patient cooperation and comprehension are extremely important to ensure predictable results.

### 1.2. Recipient Site Criteria

The most important criteria for success involving the recipient site is adequacy of bone support. There must be sufficient alveolar bone support in all dimensions with adequate attached keratinized tissue to allow for stabilization of the transplanted tooth. In addition, the recipient site should be free from acute infection and chronic inflammation.

### 1.3. Donor Tooth Criteria

The donor tooth should be positioned such that extraction will be as atraumatic as possible. Abnormal root morphology, which makes the tooth removal difficult and may involve tooth sectioning, is contraindicated for this surgery. Teeth with either open or closed apices may be donors; however, the most predictable results are obtained with teeth showing one-half to two-thirds of root development. Surgical manipulation of teeth with less than one-half root formation may be too traumatic and could compromise further root development causing incomplete maturation or alteration in morphology. When root development is greater than two-thirds, the increased length may cause encroachment on vital structures such as the maxillary sinus or the inferior alveolar nerve. Furthermore, a tooth with complete or near complete root formation will generally require root canal therapy, while a tooth with an open apex will remain vital and should continue root development after transplantation. In the latter case, successful transplantation without the need for further endodontic therapy is usually seen [4].

The donor tooth chosen for autotransplantation should be of limited value in the dentition, for example, a third molar. This case report presents successful transplantation of a mandibular third molar with complete root formation.

## 2. Case Report

**Figure 1:**
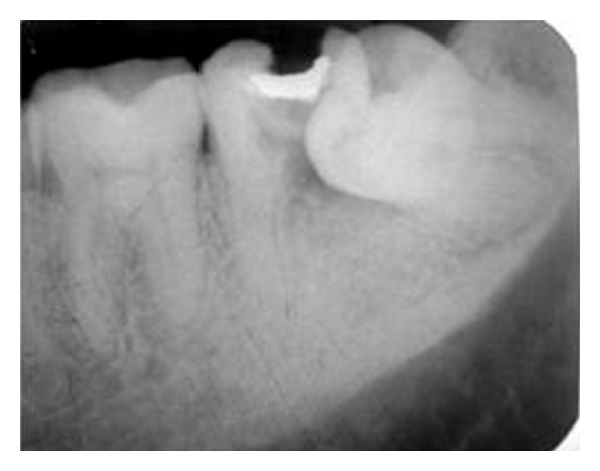
Preoperative radiograph showing 36, 37 and 38.

 A 36-year-old male patient reported to the Department of Conservative Dentistry with a chief complaint of caries with 37 and food impaction in relation to 37 and 38. Clinical and radiographic examination (Figure 1) revealed caries on the distal aspect of 37 encroaching into the pulp space with resorbed distal root and a mesioangular impaction of 38. Involved second molar tooth (37) was nonrestorable and was indicated for extraction. Impacted third molar 38 was a sound, mature tooth without caries and was found to be ideal for transplantation. The mesiodistal dimensions of 37 and 38 were suitable for transplantation.

**Figure 2:**
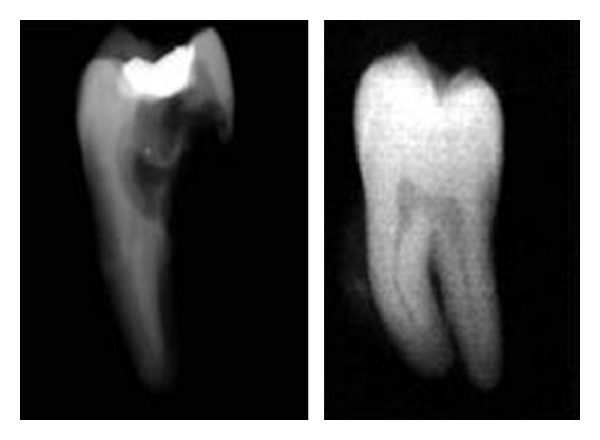
Extracted teeth 37, 38.

**Figure 3:**
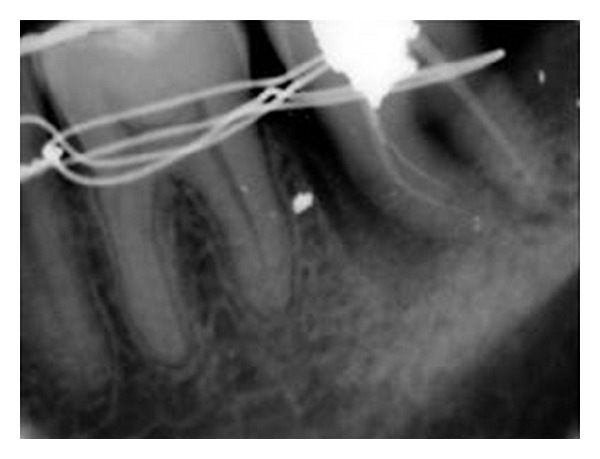
After transplantation and splinting.

After taking the complete medical history and explaining the risks and benefits of the procedure to the patient, an informed consent was taken. Patient was prescribed 1000 mg Amoxycillin one hour before surgery to prevent infection and 400 mg of Ibuprofen was prescribed to prevent post-treatment pain. 37 and 38 were extracted atraumatically (Figure 2) without damaging the buccal and lingual cortical plates. Root canal therapy for 38 was completed extra-orally within 30 minutes using ProTaper NiTi rotary files. Care was taken to prevent any damage to periodontal ligament (PDL) cells by holding the tooth at cementoenamel junction, and the roots were kept moist with sterile gauze soaked in coconut water during access cavity preparation. During biomechanical preparation and obturation of the root canals, the roots were wrapped with sterile gauze and were submerged in coconut water in a sterile dappen dish. Access cavity was restored with high copper silver amalgam. Alveolar socket of 37 was widened using surgical bur in a slow speed hand piece, under copious saline irrigation and 38 was transplanted into 37 socket. Suturing was performed with silk thread to stabilize the soft tissues and the transplanted tooth. Nonrigid intraradicular occlusal splinting with malleable orthodontic wire and composite resin was done from 35 to 37 (Figure 3). Postoperative IOPA radiograph revealed radioopaque object in the interdental area of 36 and transplanted tooth. This could be a fragment of amalgam restoration of 37 that may got impregnated into the tissues during intraradicular groove preparation for splinting. The patient was instructed to perform daily mouth rinsing with 0.12% Chlorhexidine gluconate, twice a day for seven days, Amoxicillin 500 mg thrice daily, Metronidazole 400 mg thrice a day, and Etrobax 120 mg twice daily for 5 days were prescribed. 

**Figure 4:**
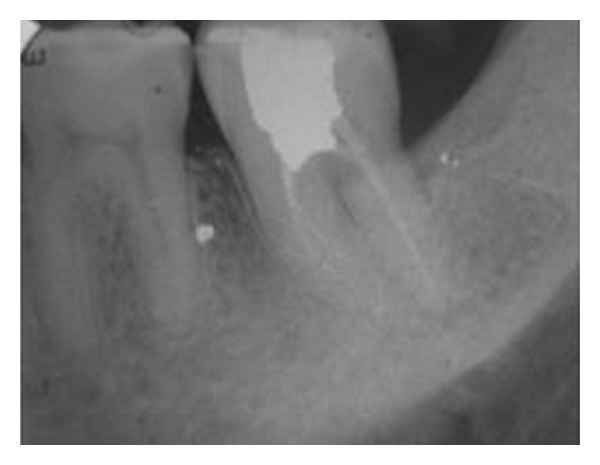
1-year recall radiograph.

 Sutures were removed after a week and healing appeared satisfactory. The patient was reviewed after 1 week, 1 month, 2 months, 6 months, and 1 year. Non rigid splinting was removed after 2 months. Patient was asymptomatic during recall visits. Clinical examination demonstrated absence of mobility and masticatory dysfunction.The occlusion was found to be normal. There were no signs of loss of attachment. Percussion test did not produce the characteristic metallic sound of ankylosis. At the 1-year followup, radiographic examination revealed healing of the site (Figure 4). There were no signs suggestive of root resorption or other pathological processes and revealed normal periodontal attachment. At the end of 1 year, bone growth around the transplanted tooth was considered satisfactory. 

## 3. Discussion

 Patient was insistent on maintaining the number of teeth in his arch as he is a diver by profession in navy. Other treatment option for this case was to extract 37 and 38 followed by the placement of an implant. But due to the expenses of implant therapy, and due to local limitations like the remaining bone support and close proximity to the anatomical structure, autotransplantation was selected as treatment option. 

 The success rate of autogenous tooth transplantation in the 1950s was approximately 50% because of the difficulty in predicting root development after transplantation and dental root resorption [5, 6]. Because too little was known of the causes and prevention of root resorption of transplanted autogenous teeth, the procedure was used infrequently. Since 1990, many studies have evaluated the healing of periodontal tissues and the incidence of dental root resorption after transplantation and the transplant success rate has increased rapidly, drawing new clinical interest [7–9]. Tsukiboshi [7] reported a 90% survival rate and an 82% success rate in 250 cases observed for 6 years. Lundberg and Isaksson [8] reported a success rate of 94% in cases with incompletely formed roots and 84% in cases with completely formed roots, whereas Mejàre et al. [9] reported a high success rate for cases with mature teeth. 

 Like other surgical procedures, careful case selection and treatment planning is essential for successful autotransplantation. The donor tooth and recipient site should be both examined meticulously for suitability and appropriate dimensions [4, 10]. The recipient site should have adequate bone support with sufficient attached keratinized tissue to allow tooth stabilization and be free of infection and/or inflammation [4].

 Insufficient buccolingual width in the recipient site or excessive preparation of the site may result in resorption of the alveolar ridge, loss of buccal bone coverage, and consequent loss of periodontal integrity. An extensive study that evaluated autotransplantation of 53 molar teeth with developed roots reported that lack of buccal bone plate was the only significant predictor for transplant failure [9].

 In the present case, occlusal reduction of the transplanted tooth was done to protect the tooth from any occlusal trauma and to allow proper healing of the periradicular tissues. As the bone support in the recipient site was poor, a physiological splint was kept in place for 2 months without having the risk of ankylosis.

Preservation of the periodontal ligament (PDL) cells is critical for the success of a tooth transplant [11–14]. Accordingly, teeth which have sharp root curvatures are not good candidates for transplantation because there is an increased risk of PDL damage and cemental tear during extraction [1, 10]. Extraction procedure was carried out with special care and to preserve periodontal ligament cells of third molar, the roots were wrapped with gauze soaked in coconut water. 

The periodontal ligament cells are extremely sensitive, and their survival ability is significantly reduced if the extraoral dry time is prolonged [15]; this effect has been demonstrated to be significant after 18 minutes [12, 16]. The increased length of the extra-alveolar time increases the possibility of inflammatory replacement resorption, reduces the healing capability of the periodontal ligament cells which in turn induces unfavourable consequences such as inflammatory external root resorption. In the present case, inspite of keeping 30 minutes of extra alveolar time, there was no external root resorption that may be due to coconut water used for maintaining the viability of the cells. Coconut water is an excellent storage media for avulsed teeth, as it has a P^H^ and osmolality compatible to PDL cells [14].

 After autotransplantation of permanent teeth with fully developed roots pulpal revascularization or revitalization is not expected [17, 18]. Previous studies have shown a high incidence of pulp necrosis in mature teeth. Therefore, root canal therapy or root resection with retrograde filling has been advised to prevent pulpal infection and/or inflammation and subsequent root resorption [19–21], Routinely, root canal therapy is performed a few weeks after transplantation [21]. However, Bae et al. recommend that if the donor tooth is easily accessed, root canal therapy should be performed before transplantation [15]. In this case, root canal therapy was done extraorally due to limited accessibility and presence of mesial root curvature of the donor tooth that may hamper the effective endodontic treatment.

 Effective and successful transplantation requires approximation between shape and size of the donor tooth and the receptor site. It also has been postulated that maxillary transplants have greater risk of failure due to the wide variation in the size and shape of the teeth and the proximity of the maxillary antrum to the molar sockets [15]. However in this case, the tooth to be transplanted fitted the recipient site; thus, the procedure caused minimal trauma. In addition, the favorable cervical adaptation between the tooth and bone decreased the chance of infection and increased the likelihood of an uneventful healing and periodontal reattachment. The healing was satisfactory inspite of presence of amalgam fragment between 36 and the transplanted tooth.

 It can be assumed that the physiologic mobility of the transplanted tooth stimulated the activation of periodontal ligament cells (i.e., fibroblasts, cementoblasts and osteoblasts) and bone repair [5]. The transplanted molar tooth in the present case had complete developed roots, that encouraged transplantation in these teeth. Both clinical, and radiographic outcomes were considered satisfactory after 1 year.

 Complications of autogenous tooth transplant include root resorption and attachment loss, and its success rate is lower than implant prosthesis. Nonetheless, autogenous teeth transplantation results in good utilization, the maintenance and regeneration of alveolar bone, and the maintenance of attached gingiva with a natural shape. 

## 4. Conclusions 

 There is obvious limitation in terms of versatility in the application of transplantation versus implantation in replacing missing teeth. The availability of suitable size and morphology of donor tooth are the major constraint. The success rate of implant is also higher than that of transplant. Reported survival rates of autotransplantation vary from 74–100%, artificially drilled recipient sockets tend to be at the lower range of success rate. With appropriate patient selection, and presence of a suitable donor tooth and recipient site, autogenous transplantation should be considered as a viable option for avoiding/prevention of an edentulous space.
